# The zebrafish ETS transcription factor Fli1b functions upstream of Scl/Tal1 during embryonic hematopoiesis

**DOI:** 10.1242/bio.061948

**Published:** 2025-04-09

**Authors:** Valentina Laverde, Luiza Loges, Saulius Sumanas

**Affiliations:** Department of Pathology and Cell Biology, USF Health Heart Institute, University of South Florida, Tampa, FL 33602, USA

**Keywords:** Zebrafish, Hemangioblast, Blood, Erythroid, Myeloid, Red blood cell, Macrophage, Neutrophil, Fli1

## Abstract

During embryonic development, vascular endothelial and hematopoietic cells are thought to originate from a common precursor, the hemangioblast. The evolutionarily conserved ETS transcription factor FLI1 has been previously implicated in hemangioblast formation and hematopoietic and vascular development. However, its role in regulating the hemangioblast transition into hematovascular lineages is still incompletely understood. Its zebrafish paralog Fli1b (also known as Fli1rs) functions partially redundantly with the ETS transcription factor Etv2 (also known as Etsrp) during vasculogenesis and angiogenesis. However, its role in embryonic hematopoiesis has not been previously investigated. Here, we show that zebrafish *fli1b* mutants have a reduced formation of primitive erythrocytes and hematopoietic stem and progenitor cells, and display reduced expression of key regulators of hematopoiesis, including *scl* (also known as *tal1*), *gata1a* and *runx1.* Expression of *scl* was sufficient to partially rescue defects in erythroid differentiation in *fli1b* mutants, arguing that *scl* functions downstream of *fli1b* during primitive erythropoiesis. In addition, myelopoiesis was strongly misregulated in *fli1b* mutants. Although the formation of the earliest myeloid progenitors – neutrophils and macrophages – was greatly reduced in *fli1b* mutants, this was compensated by the increased emergence of myeloid cells from the alternative hematopoietic site – the endocardium. Intriguingly, myeloid cells in *fli1b* mutants retained vascular endothelial marker expression, suggesting that they are present in a hemangioblast-like state. In summary, our results demonstrate a novel role of *fli1b* transcription factor in regulating embryonic hematopoiesis.

## Teaser

**Summary:** This study investigates the role of the ETS transcription factor Fli1b in regulating hematopoiesis in zebrafish embryos. *fli1b* mutants show reduced erythropoiesis and myelopoiesis, which is compensated by increased myeloid marker expression at the alternative hematopoietic site, the endocardium.

## INTRODUCTION

The earliest vascular endothelial and hematopoietic cell lineages in different vertebrates are thought to originate from a common precursor, the hemangioblast (Lacaud and Kouskoff, 2017). In mammalian embryos, the putative hemangioblasts emerge in the yolk blood islands and give rise to primitive erythrocytes, megakaryocytes and macrophages, as well as vascular endothelial cells (Biben et al., 2023). To date, the molecular mechanisms that regulate hemangioblast differentiation into the earliest hematopoietic and endothelial lineages are still incompletely understood.

Although it is difficult to study the earliest steps in blood and vascular differentiation in mammalian embryos, zebrafish has emerged as an advantageous embryonic model. The molecular mechanisms regulating hematovascular development are highly conserved among zebrafish and mammalian embryos. Blood and vascular progenitors in zebrafish embryos emerge during early somitogenesis stages from the lateral plate mesoderm (LPM), which is considered to be equivalent to mammalian blood islands. The anterior LPM (ALPM) gives rise to cranial vasculature and the primitive myeloid lineages, which include macrophages and neutrophils, whereas the posterior LPM (PLPM) gives rise to erythroid cells as well as trunk and tail vasculature (Davidson and Zon, 2004; Warga et al., 2009). Cell lineage-tracing studies have indicated that many (but not all) endothelial and hematopoietic cells in zebrafish share a common progenitor, the hemangioblast (Vogeli et al., 2006). However, these lineages separate very early and, to the best of our knowledge, cells that co-express vascular endothelial and hematopoietic markers have not been observed *in vivo* during later stages of embryogenesis, with the exception of the hemogenic endothelium that gives rise to hematopoietic stem cells (HSCs) (Bertrand et al., 2010; Kissa and Herbomel, 2010).

Several ETS transcription factors have been implicated in regulating distinct steps during hematopoietic and endothelial differentiation. The ETS transcription factor friend leukemia integration 1 (Fli1) functions near the top of the transcriptional cascade and is involved in regulating both vascular endothelial and hematopoietic differentiation (Ben-David et al., 2022). *Fli1-*deficient murine embryos die due to hemorrhages around embryonic day (E) 11.5. They show deficient vascular endothelial differentiation as well as defects in hematopoiesis, mainly in the block of megakaryocyte differentiation (Abedin et al., 2014; Hart et al., 2000; Spyropoulos et al., 2000). However, the role of Fli1 in the formation of primitive erythroid and myeloid lineages is less clear. Erythrocytes appear to be present at E8.5 and E9.5 in *Fli1* mutant embryos. Although greatly reduced fetal liver hematopoiesis has been reported, this could be a consequence of hemorrhages and HSCs failing to repopulate the fetal liver (Spyropoulos et al., 2000). Overexpression of Fli1 in EWS-Fli1 fusion protein inhibits erythroid differentiation (Pereira et al., 1999; Tamir et al., 1999). Low levels of Fli1 expression in myelo-erythroid progenitors are thought to promote erythropoiesis, whereas high levels promote megakaryocyte differentiation (Ben-David et al., 2022). In contrast, inhibition of Fli1 function in frog (*Xenopus laevis*) embryos using morpholinos (MOs) results in the inhibition of hemangioblast differentiation and the downregulation of primitive and definitive hematopoiesis (Liu et al., 2008). However, these results have not been validated in genetic mutants.

Due to genomic duplication, there are two homologs of mammalian *Fli1* present in zebrafish, *fli1a* (also known as *fli1*) and *fli1b* (also known as *fli1rs*), both of which are expressed in vascular endothelial cells (Brown et al., 2000; Craig et al., 2015). In addition, *fli1a* is expressed in hematopoietic progenitors (Brown et al., 2000). Overexpression of Fli1a and transactivating domain fusion protein was found to cause expansion of hematopoietic and vascular markers in zebrafish embryos (Liu et al., 2008). MO knockdown of Fli1a together with Erg or other ETS transcription factors caused defects in angiogenesis (Liu and Patient, 2008; Pham et al., 2007). However, no hematopoietic defects have been previously reported in Fli1a-deficient embryos, and previous MO knockdown results have not been validated using genetic mutants.

We have previously reported characterization of the zebrafish *fli1b* mutant, which was isolated in a transposon-mediated insertional mutagenesis screen (Craig et al., 2015). *fli1b* homozygous mutants were viable and did not show any obvious defects in vascular development. However, they exhibited a partial redundancy with another ETS transcription factor, Etv2 (also known as Etsrp), which functions as a master regulator of vasculogenesis (Sumanas and Lin, 2006). *etv2* mutants showed loss of early vascular differentiation, yet vasculogenesis partially recovered during the 24-72 h post fertilization (hpf) stages (Craig et al., 2015). This recovery was completely absent in *etv2; fli1b* double-deficient embryos, illustrating a partial redundancy between these two factors (Craig et al., 2015). However, the role of *fli1b* in hematopoiesis has not been previously investigated.

In this study, we analyzed hematopoietic defects in *fli1b* mutant embryos. We show that primitive erythropoiesis and HSC formation were greatly reduced in *fli1b* mutant embryos. In addition, formation of primitive myeloid progenitors – macrophages and neutrophils – was significantly diminished in *fli1b* mutants. In contrast, *fli1b* mutant embryos showed expansion of myeloid cells derived from an alternative hematopoietic site, the endocardium. Intriguingly, these cells co-expressed both myeloid and vascular endothelial markers in *fli1b* mutants, suggesting that they are present in a hemangioblast-like state. These results identify new roles for *fli1b* in regulating hematopoietic differentiation and will be important for our understanding of the molecular mechanisms and transcriptional pathways that regulate hemangioblast and vascular differentiation during development and regeneration.

## RESULTS

### Primitive and definitive hematopoiesis are greatly reduced in *fli1b^−/−^* zebrafish embryos

**Fig. 1.**
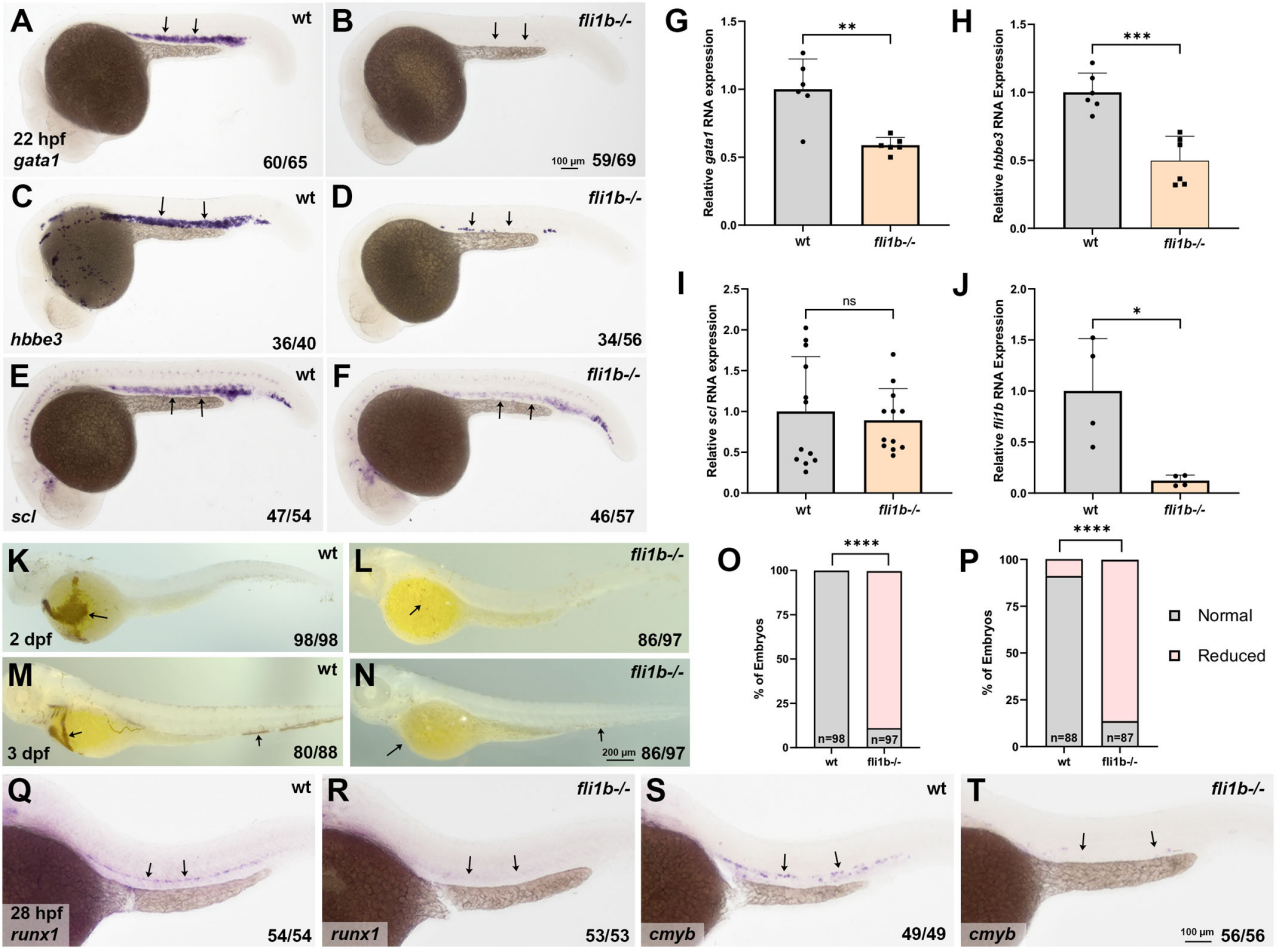
***fli1b* mutants display reduced primitive and definitive hematopoiesis.** (A-F) Whole-mount *in situ* hybridization (WISH) analysis of expression of the erythroid markers *gata1* (A,B) and *hbbe3* (C,D) and the hematopoietic marker *scl* (E,F) in *fli1b^−/−^* mutant and wild-type (wt) embryos (in *fli1a:GFP* background) at 22 h post fertilization (hpf). *fli1a:GFP* embryos were used as controls to enable fluorescence analysis of any potential defects in vascular development in *fli1b^−/−^* embryos, which show GFP expression linked to *fli1b* mutation. Note greatly reduced expression of *gata1* and *hbbe3* and moderately reduced expression of *scl* in the intermediate cell mass region (arrows) in *fli1b* mutants. (G-J) qPCR analysis of *gata1* (G), *hbbe3* (H), *scl* (I) and *fli1b* (J) expression in *fli1b^−/−^* mutant and wt *fli1a:GFP* embryos at the 20-somite stage. Note the significant reduction in *gata1*, *hbbe3* and *fli1b* expression in *fli1b* mutant embryos. Bars show mean±s.d. ns, not significant; *P<0.05; **P<0.01; ***P<0.001; Student's two-tailed unpaired *t*-test. (K-N) Heme staining using o-dianisidine in *fli1b* mutant and control wt *fli1a:GFP* embryos at 2 and 3 days post fertilization (dpf). Note greatly reduced staining (arrows) in *fli1b* mutant embryos. (O,P) Quantification of embryos with normal and reduced heme staining at 2 dpf (O) and 3 dpf (P). ****P<0.0001; Fisher's exact test. (Q-T) WISH analysis for expression of the hematopoietic stem cell and progenitor markers *runx1* and *cmyb* at 28 hpf in the trunk region of *fli1b* mutant and control wt *fli1a:GFP* embryos. Note the greatly reduced or absent staining (arrows) in *fli1b* mutants. All experiments have been replicated at least twice.

To test whether *fli1b* is required for primitive hematopoiesis, we analyzed the expression of hematopoietic markers in *fli1b^−/−^* mutant and wild-type (wt) control *fli1a:GFP* embryos using whole-mount *in situ* hybridization (WISH). The expression of the erythroid markers *gata1a* (hereafter *gata1*) and *hbbe3* in wt embryos was present in the intermediate cell mass region above the yolk extension at 22 hpf (Fig. 1A,C). In *fli1b^−/−^* mutants, the expression of *gata1* was strongly reduced in 85.5% of embryos, whereas *hbbe3* expression was reduced in 60.7% of embryos at 22 hpf (Fig. 1A-D). The transcription factor *stem cell leukemia* (*scl*, also known as *tal1*) is considered a master regulator of early hematopoiesis and functions upstream of *gata1* and *hbbe3* (Dooley et al., 2005; Juarez et al., 2005; Patterson et al., 2005). *scl* expression in the intermediate cell mass was reduced in 80.7% of *fli1b^−/−^* embryos at 22 hpf, although the reduction was not as significant as that of *gata1* and *hbbe3* (Fig. 1E,F). To confirm WISH results, we performed quantitative PCR (qPCR) for *gata1*, *hbbe3* and *scl* using wt and *fli1b^−/−^* embryos at the 20-somite stage*.* Expression of both *gata1* and *hbbe3* was significantly reduced in *fli1b^−/−^* zebrafish embryos, whereas *scl* expression was not strongly affected (Fig. 1G-I), possibly due to its early expression in both red blood cell (RBC), vascular endothelial cell and neuronal cell populations. As expected, *fli1b* mRNA expression was greatly reduced in *fli1b^−/−^* embryos based on qPCR analysis (Fig. 1J). We then performed heme staining using o-dianisidine to evaluate the presence of RBCs in *fli1b^−/−^* zebrafish embryos at 2 and 3 days post fertilization (dpf). Heme staining in *fli1b^−/−^* embryos was significantly reduced at both time points compared to wt control *fli1a:GFP* embryos (Fig. 1K-P). Altogether, these results indicate that primitive erythropoiesis is diminished in *fli1b* mutant embryos.

To determine whether *fli1b* mutants also showed defects in definitive hematopoiesis, WISH was performed at 28 hpf for the hematopoietic stem and progenitor cell (HSPC) markers *runx1* and *cmyb* (also known as *myb*). Expression of both *runx1* and *cmyb* in HSPCs, located along the wall of the dorsal aorta, was substantially reduced in *fli1b^−/−^* embryos compared to that in wt controls (Fig. 1Q-T). These results argue that both primitive and definitive hematopoiesis are deficient in *fli1b^−/−^* embryos.

### *fli1b* expression is restricted to vascular endothelial cells

**Fig. 2.**
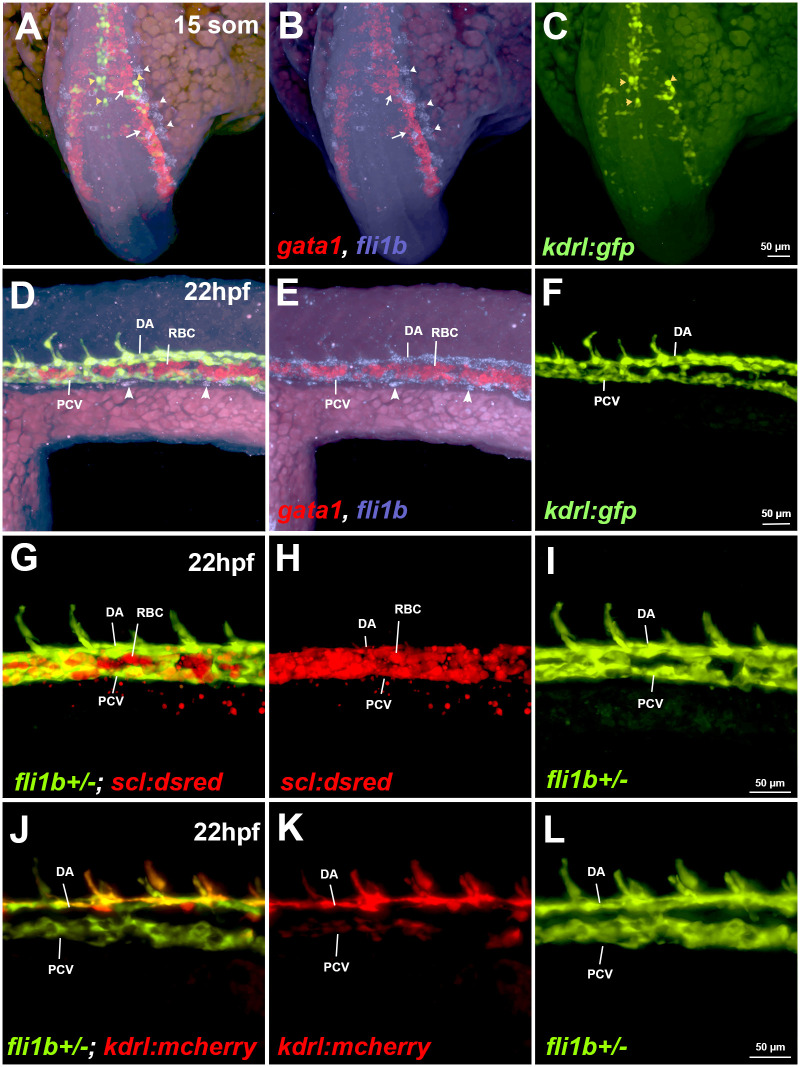
**Analysis of *fli1b* expression in vasculature and red blood cells.** (A-F) Expression analysis of *fli1b*, the erythroid marker *gata1* and the vascular endothelial marker *kdrl:GFP* at 15-somite (som) and 22-hpf stages. Fluorescence *in situ* hybridization (FISH) for *fli1b* (magenta) and *gata1* (red) was performed using hybridization chain reaction (HCR) on *kdrl:GFP* zebrafish embryos. Note *gata1* expression in red blood cells (RBCs; white arrows, A,B), and *fli1b* and *kdrl:GFP* co-expression in vascular endothelial cells at the 15-somite stage (yellow arrowheads, A,C), and in the dorsal aorta (DA) and posterior cardinal vein (PCV) at 22 hpf (D-F). Early vascular endothelial progenitor cells (white arrowheads, A,B), as well as late-forming vascular progenitors (white arrowheads, D,E) were positive for *fli1b* expression but negative for *kdrl:GFP.* Note that RBCs were largely negative for *fli1b* expression. (G-L) Live imaging of fluorescent *fli1b^+/−^; scl:dsRed* or *kdrl:mCherry* embryos at 22 hpf. Due to the gene-trap construct insertion into the *fli1b* locus, GFP fluorescence is indicative of *fli1b* expression. Note the absence of *fli1b* expression (green) in the RBCs, positive for *scl:dsRed*, whereas it overlaps with vascular endothelial *kdrl:mCherry* expression. Also note that *scl:dsRed* expression labels both vascular endothelial cells (positive for *fli1b*) and erythrocytes (negative for *fli1b*). Images represent ten embryos (A-C), nine embryos (D-I) and six embryos (J-L).

We have previously demonstrated that *fli1b* is expressed in vascular endothelial cells (Craig et al., 2015). To clarify whether *fli1b* is also expressed in hematopoietic cells or their progenitors, we analyzed co-expression of the erythroid progenitor marker *gata1* and *fli1b* in vascular endothelial *kdrl:GFP* embryos at the 15-somite and 22 hpf stages using the hybridization chain reaction (HCR) (Fig. 2A-F). However, no overlap between *gata1* expression in RBCs and *fli1b* expression was observed. In contrast, *kdrl:GFP* and *fli1b* expression overlapped extensively in vascular endothelial cells at 22 hpf (Fig. 2D-F). Notably, *fli1b* expression only had a partial overlap with *kdrl:GFP* expression at the 15-somite stage, likely due to strong *fli1b* expression in vascular endothelial progenitors (angioblasts), which are negative for *kdrl:GFP* (Fig. 2A-C)*.* In addition, we analyzed *fli1b* and *scl* co-expression in live *fli1b^+/−^; scl:dsRed* embryos. Because of the gene-trap insertion in the *fli1b* locus, GFP expression labels *fli1b-*positive cells in heterozygous *fli1b^+/−^* embryos, whereas *scl:dsRed* expression is apparent in blood and endothelial cells. At the 22 hpf stage, there was no apparent overlap between GFP and *scl:dsRed* expression in blood cells or their progenitors, whereas GFP and *scl:dsRed* expression overlapped in vascular endothelial cells (Fig. 2G-I). Similarly, GFP and *kdrl:mCherry* expression overlapped in vascular endothelial cells in *fli1b^+/−^; kdrl:mCherry* embryos at the 22 hpf stage (Fig. 2J-L). Altogether, these results confirm that *fli1b* expression is restricted to vascular endothelial cells and their progenitors and is largely absent from erythroid cells.

### *scl* functions downstream of *fli1b* during primitive hematopoiesis

**Fig. 3.**
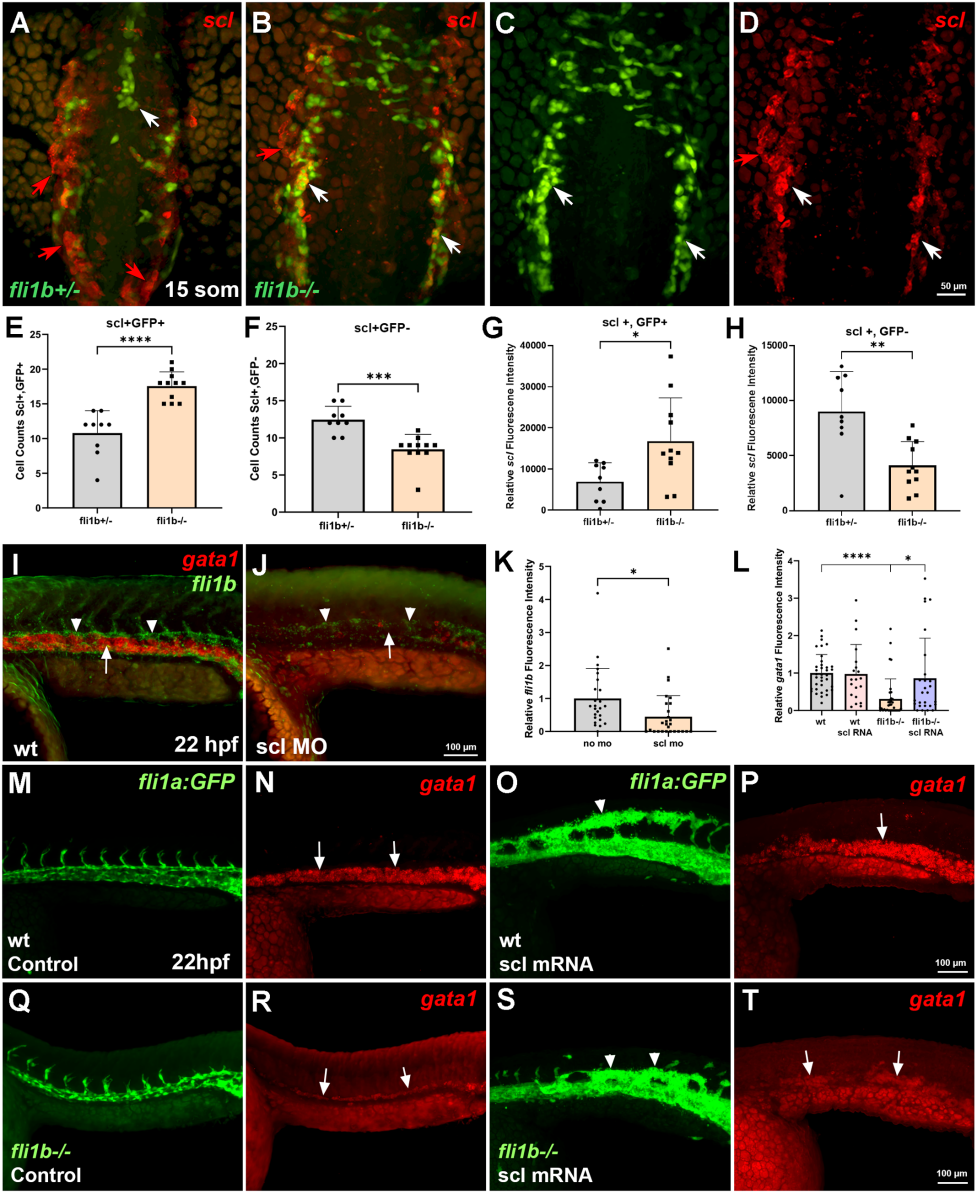
***fli1b* functions upstream of *scl* in hematopoietic progenitors.** (A-D) *scl* expression analysis using hybridization chain reaction (HCR) in the trunk region of *fli1b^+/−^* and *fli1b^−/−^* embryos at the 15-somite stage. Bilaterally located hematopoietic progenitors in *fli1b^+/−^* embryos show high *scl* and no GFP expression (*scl*^+^ GFP^−^, red arrows, A), whereas vascular endothelial progenitors, which are either at the midline or in the process of migration, show high GFP and low *scl* (*scl^low^* GFP*^+^*, white arrow, A). In *fli1b^−/−^* embryos, the number of hematopoietic *scl^+^* GFP*^−^* cells is reduced (red arrows, B-D), whereas the number of double-positive *scl^+^* GFP*^+^* cells is increased. Many double-positive cells are located bilaterally (white arrows, B-D), where hematopoietic cells are positioned in the control *fli1b^+/−^* embryos, suggesting that some hematopoietic cells have a mixed identity in *fli1b^−/−^* embryos. (E-H) Quantification of cell number and *scl* fluorescence intensity in the trunk and tail region of *fli1b^+/−^* and *fli1b^−/−^* embryos at the 15-somite stage. Note the increased *scl^+^* GFP*^+^* cell number and higher *scl* fluorescence intensity of in *fli1b^−/−^* embryos, possibly due to increased *fli1b* and GFP expression in hematopoietic cells, which are GFP negative in control *fli1b^+/−^* embryos. *scl^+^* GFP*^–^* hematopoietic cell number and *scl* expression are reduced in *fli1b^−/−^* mutants, indicative of reduced erythroid cell differentiation. *P<0.05; **P<0.01; ***P<0.001; ****P<0.0001; Student's two-tailed unpaired *t*-test. (I-K) HCR analysis for erythroid *gata1* (arrows) and vascular endothelial *fli1b* (arrowheads) expression, which is reduced in the trunk region of *scl* MO-injected embryos compared to that in uninjected controls at the 22 hpf stage. *P<0.05; Student's two-tailed unpaired *t*-test. (L-T) *scl* mRNA rescues *gata1* expression in *fli1b* mutant embryos at 22 hpf stage. (L) Quantification of *gata1* fluorescence in the trunk region. *P<0.05; ****P<0.0001, Student's two-tailed unpaired *t*-test. HCR analysis of *gata1* expression in control uninjected or *scl* mRNA-injected wt *fli1a:GFP* (M-P) or *fli1b^−/−^* (Q-T) embryos. Note expansion of *fli1a:GFP* (arrowhead) and *gata1* expression (arrows) in wt embryos injected with *scl* mRNA (O,P). Uninjected *fli1b^−/−^* embryos showed reduced *gata1* expression (arrows, R), which was expanded in *scl* mRNA-injected embryos (arrows, T). Similar regions of the embryonic mid-trunk are shown in M-T. Bars in E-H,K,L show mean±s.d.

*scl* is one of the earliest factors expressed in hematopoietic progenitors. It functions upstream of *gata1* in inducing erythroid differentiation. In addition, *scl* is expressed in vascular endothelial progenitors and is required for endothelial differentiation (Dooley et al., 2005; Juarez et al., 2005; Patterson et al., 2005). To test whether *scl* expression was affected in vascular endothelial or hematopoietic progenitors in *fli1b* mutant embryos, we analyzed its expression in *fli1b^+/−^* and *fli1b^−/−^* zebrafish embryos at the 15-somite stage. At least two distinct *scl^+^* cell populations could be identified in the trunk region of *fli1b^+/−^* embryos at this stage: low *scl*, high GFP (*scl^low^* GFP^+^) cells, which presumably correspond to vascular endothelial cells, and high *scl* single-positive cells (*scl^+^* GFP*^−^*), which are presumptive hematopoietic progenitors (Fig. 3A). Notably, at this stage, hematopoietic (*scl^+^* GFP^−^) cells were located bilaterally, whereas most endothelial cells were either positioned at the midline or were in the process of migrating towards the midline (Fig. 3A). Intriguingly, the number of hematopoietic (*scl^+^* GFP^−^) cells was significantly reduced in *fli1b*^−/−^ embryos. In contrast, the number of *scl^+^* GFP*^+^* cells was increased in *fli1b*^−/−^ mutants (Fig. 3B-F). However, these double-positive cells did not migrate to the midline, where vascular endothelial cells were located, but were positioned bilaterally, similar to hematopoietic progenitors in *fli1b^+/−^* embryos (Fig. 3B-D). These results suggest that some cells have a mixed identity and co-express hematopoietic and vascular endothelial markers in *fli1b^−/−^* mutants.

In *fli1b^+/−^* embryos, bilaterally located hematopoietic cells showed strong *scl* expression, whereas endothelial cells had much lower *scl* expression (Fig. 3A). The hematopoietic cells (*scl^+^* GFP*^−^*) showed greatly reduced intensity of *scl* expression in *fli1b^−/−^* mutants. In contrast, bilaterally located *scl^+^* GFP*^+^* cells showed stronger *scl* expression in *fli1b^−/−^* mutants compared to that in *fli1b^+/−^* embryos (Fig. 3G,H). These results suggest that in *fli1b^−/−^* mutants, (1) *scl* expression in hematopoietic progenitors is reduced, thus resulting in defective erythropoiesis; and (2) a subset of erythroid progenitors show endothelial *fli1b* expression, resulting in mixed lineage identity.

We subsequently tested the epistatic relationship of *scl* and *fli1b* in vascular endothelial and blood cells. To test whether *scl* can regulate *fli1b* expression in vascular endothelial cells, wt embryos were injected at the single-cell stage with a previously validated *scl* MO to knock down *scl* function. As reported previously, similar to *scl* genetic mutants (Bussmann et al., 2007; Dooley et al., 2005; Juarez et al., 2005; Patterson et al., 2005), erythroid-specific *gata1* expression was strongly reduced in *scl* MO-injected embryos, compared to that in uninjected controls. Similarly, endothelial *fli1b* expression was also reduced in *scl* MO-injected embryos (Fig. 3I-K). These results suggest that *scl* is required to maintain *fli1b* expression in vascular endothelial cells.

To test whether hematopoietic defects observed in *fli1b* double mutants can be rescued by upregulation of *scl* expression, we microinjected *in vitro*-synthesized *scl* mRNA into control wt *fli1a:GFP* and *fli1b^−/−^* embryos and assayed for *gata1* expression at 22 hpf using HCR. Wt embryos injected with *scl* mRNA showed ectopic expansion of both *gata1* and GFP expression (Fig. 3L-P). Injected *fli1b^−/−^* embryos also showed increased *gata1* expression compared to that in uninjected *fli1b^−/−^* control embryos (Fig. 3L,Q-T). Thus, *scl* overexpression can partially rescue hematopoietic defects in *fli1b^−/−^* embryos.

### *fli1b^−/−^* mutants show deficient myelopoiesis

**Fig. 4.**
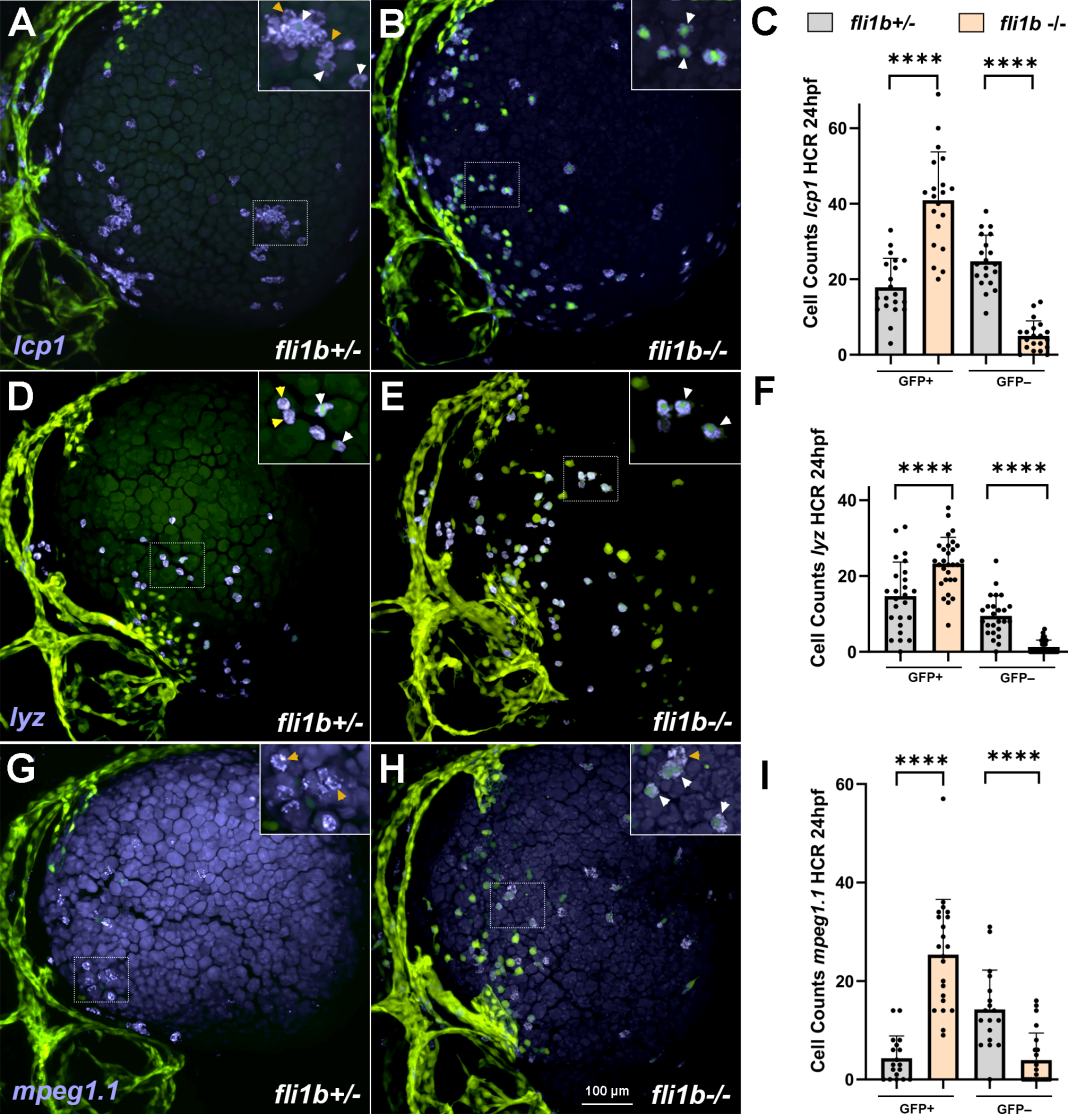
**Myeloid marker expression analysis at 24 hpf.** HCR analysis for expression of the pan-myeloid marker *lcp1* (A-C), the neutrophil marker *lyz* (D-F) and the macrophage marker *mpeg1.1* (G-I) in *fli1b^+/−^* control and *fli1b^−/−^* mutant embryos. Two populations of *lcp1^+^* GFP*^+^* (or *lyz^+^* GFP*^+^* or *mpeg1.1^+^* GFP*^+^*) and *lcp1^+^* GFP*^−^* (or *lyz^+^* GFP*^−^* or *mpeg1.1^+^* GFP*^−^*) were observed. Boxed areas are shown at a higher magnification in the upper right inserts. White arrowheads show GFP^+^ myeloid cells, whereas yellow arrowheads show GFP^−^ myeloid cells. Only myeloid cells located at the yolk surface were quantified. Note that the number of GFP^+^ myeloid cells was significantly increased in *fli1b^−/−^* embryos, whereas the number of GFP^−^ myeloid cells was reduced. Myeloid cell GFP expression was faint in *fli1b^+/−^* embryos and much brighter in *fli1b^−/−^* embryos; both weak and strong GFP^+^ cells were included in GFP cell counts. Bars show mean±s.d. ****P<0.0001; Student's two-tailed unpaired *t*-test.

In addition to PLPM-derived erythrocytes, primitive hematopoietic lineages also include myeloid cells, such as macrophages and neutrophils, which are known to originate in the ALPM (Davidson and Zon, 2004; Warga et al., 2009). To test whether myeloid cells were affected in *fli1b^−/−^* embryos, we analyzed expression of the pan-myeloid marker *lcp1*, the macrophage marker *mpeg1.1* and the neutrophil marker *lyz* at 24 hpf. We noticed two populations of *lcp1^+^* myeloid cells along the surface of the yolk of *fli1b^+/−^* embryos. 41.9% of *lcp1^+^* cells were positive for GFP expression, whereas the remaining 58.1% were negative for GFP (Fig. 4A,C). Interestingly, the number of GFP-negative (*lcp1^+^* GFP*^−^*) cells was reduced in *fli1b^−/−^* embryos, whereas the number of double-positive (*lcp1^+^* GFP*^+^*) cells was significantly increased (Fig. 4B,C). Consequently, most *lcp1^+^* cells (89.1%) were positive for GFP expression in *fli1b* mutants. In addition, GFP signals in myeloid cells were much brighter in *fli1b^−/−^* embryos, and these cells were readily apparent at the surface of the yolk (Fig. 4B). Similar to *lcp1*, two populations of *lyz^+^* and *mpeg1.1^+^* cells were observed in *fli1b^+/−^* embryos. 60.8% of *lyz^+^* and 23.4% of *mpeg1.1^+^* cells were positive for GFP expression (Fig. 4D,F,G,I). Similarly, the numbers of GFP-negative neutrophils (*lyz^+^* GFP*^−^*) and macrophages (*mpeg1.1^+^* GFP*^−^*) were greatly reduced, whereas the numbers of GFP-positive neutrophils and macrophages were significantly increased in *fli1b^−/−^* embryos (Fig. 4E,F,H,I). Thus, the majority of *lyz^+^* neutrophils (94.8%) and macrophages (86.5%) were positive for GFP expression in *fli1b* mutants at 24 hpf.

**Fig. 5.**
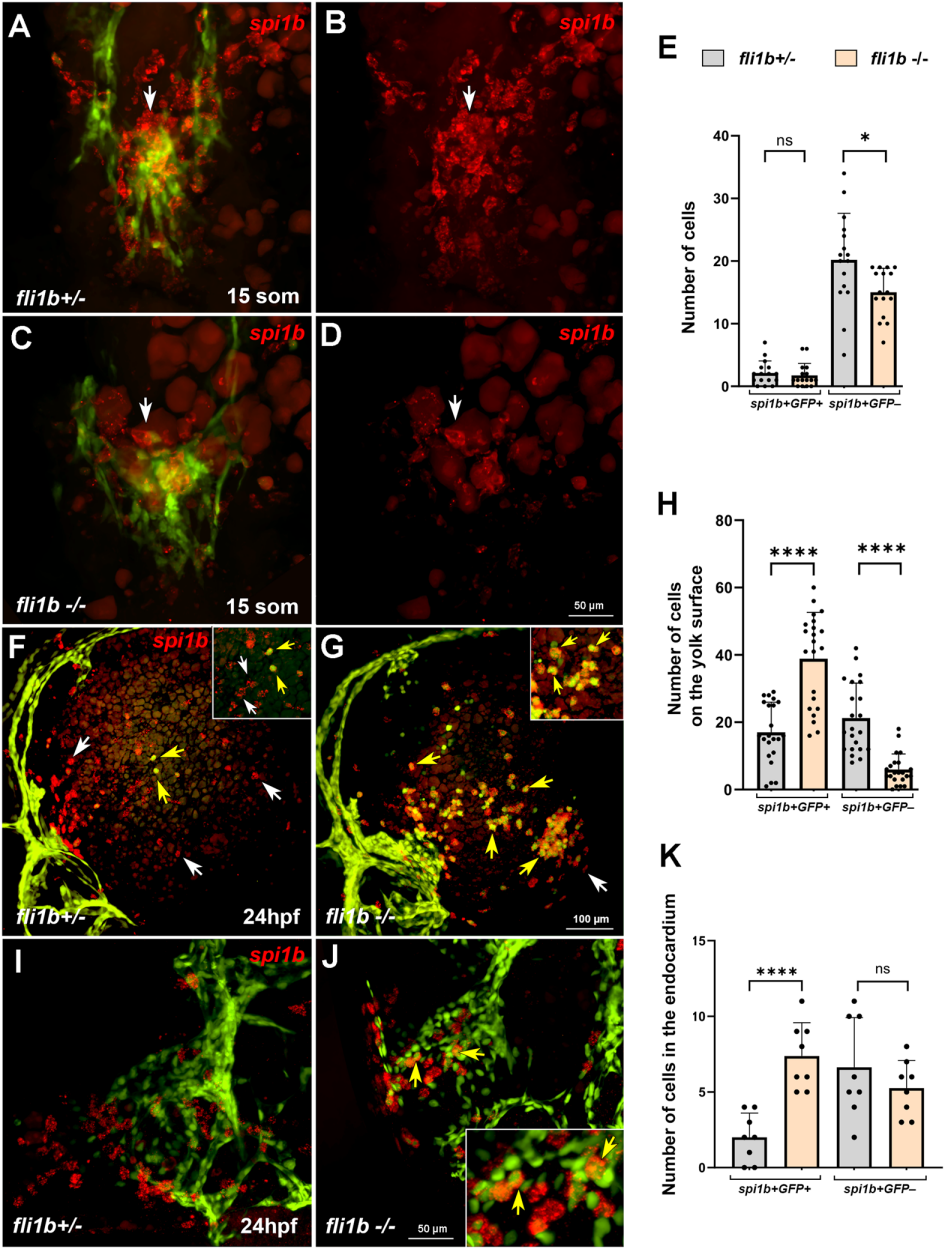
**Increased endocardial-derived myelopoiesis in *fli1b* mutant embryos.** (A-E) Hybridization chain reaction (HCR) analysis for the myeloid marker *spi1b* (red) expression in the anterior region of *fli1b^+/−^* and *fli1b^−/−^* embryos at the 15-somite stage. Merged green and red (A,C) and red-only (B,D) channels are shown; embryos were deyolked and flat mounted. Note that many myeloid progenitors are present at the midline (arrows) at this stage and do not overlap with endothelial/endocardial GFP reporter expression (some apparent overlap is an artifact of the maximum-intensity projection). *spi1b* expression was greatly reduced in *fli1b^−/−^* embryos. Some remaining autofluorescent yolk granules (dim red fluorescence) are visible in the images. The number of *spi1b^+^* cells is quantified in E. ns, not significant; *P<0.05; Student's two-tailed unpaired *t*-test. (F-H) HCR analysis for *spi1b* expression (red) at the surface of the yolk in *fli1b^+/−^* and *fli1b^−/−^* embryos at the 24 hpf stage. Note that both single-positive *spi1b^+^* GFP*^−^* cells (white arrows) and double-positive *spi1b^+^* GFP*^+^* cells (yellow arrows) are apparent in *fli1b^+/−^* embryos. The number of double-positive cells was greatly increased, whereas the number of single-positive *spi1b^+^* GFP*^−^* cells was reduced in *fli1b^−/−^* embryos, which were quantified in H. Insets in the upper right show magnified views of yolk regions in F,G. ****P<0.0001; Student's two-tailed unpaired *t*-test. (I-K) HCR analysis for *spi1b* expression in flat-mounted preparations of the endocardium of *fli1b^+/−^* and *fli1b^−/−^* embryos at 24 hpf. The number of *spi1b^+^* cells located within the endocardium (yellow arrows) was greatly increased in *fli1b^−/−^* embryos. The inset in J shows a magnified portion of the endocardium. ns, not significant; ****P<0.0001; Student's two-tailed unpaired *t*-test. Bars show mean±s.d.

In wt embryos, the earliest myeloid cells originate from the ALPM region at approximately the 10- to 15-somite stages and are largely negative for endothelial marker expression (Gurung et al., 2024). To clarify how *fli1b* affects the emergence of myeloid cells, we analyzed expression of the myeloid progenitor marker *spi1b* (also known as *pu.1*) at the 15-somite stage. As reported previously (Gurung et al., 2024), the majority of *spi1b^+^* cells were negative for GFP expression in *fli1b^+/−^* embryos at this stage, whereas only a small fraction (10.4%) were positive for GFP (Fig. 5A,B,E). *fli1b^−/−^* mutants showed a reduction in the number of *spi1b^+^* GFP*^−^* cells, whereas the small number of *spi1b^+^* GFP*^+^* cells was not changed (Fig. 5C-E).

We have recently demonstrated that the endocardium functions as a secondary hematopoietic site and gives rise to myeloid cells at the 24 to 26 hpf stage, which are initially positive for expression of the vascular endothelial/endocardial marker *kdrl* (Gurung et al., 2024). As a result, two populations of myeloid cells were distinguished, *kdrl^−^* myeloid cells derived from the early ALPM and *kdrl^+^* myeloid cells derived from the endocardium. This suggests that the GFP-positive myeloid cell population, which is expanded in *fli1b* mutants, could be derived from the endocardium. To test this, we analyzed *spi1b* expression at the surface of the yolk and in the endocardium in *fli1b^+/−^* and *fli1b^−/−^* embryos at the 24 hpf stage. Like other myeloid markers, the number of *spi1b^+^* GFP*^+^* cells at the surface of the yolk was increased, whereas the number of *spi1b^+^* GFP*^−^* cells was decreased in *fli1b^−/−^* embryos (Fig. 5F-H). Importantly, there was also an increase in the number of double-positive cells (*spi1b^+^* GFP*^+^*) in the endocardium in *fli1b^−/−^* mutants, whereas no significant change in the number of single-positive cells (*spi1b^+^* GFP*^−^*) was observed (Fig. 5I-K). This suggests that *fli1b^−/−^* mutants show reduced primitive myelopoiesis in the ALPM and increased myelopoiesis in the endocardium.

### Myeloid cells display expression of vascular endothelial markers in *fli1b^−/−^* mutants

**Fig. 6.**
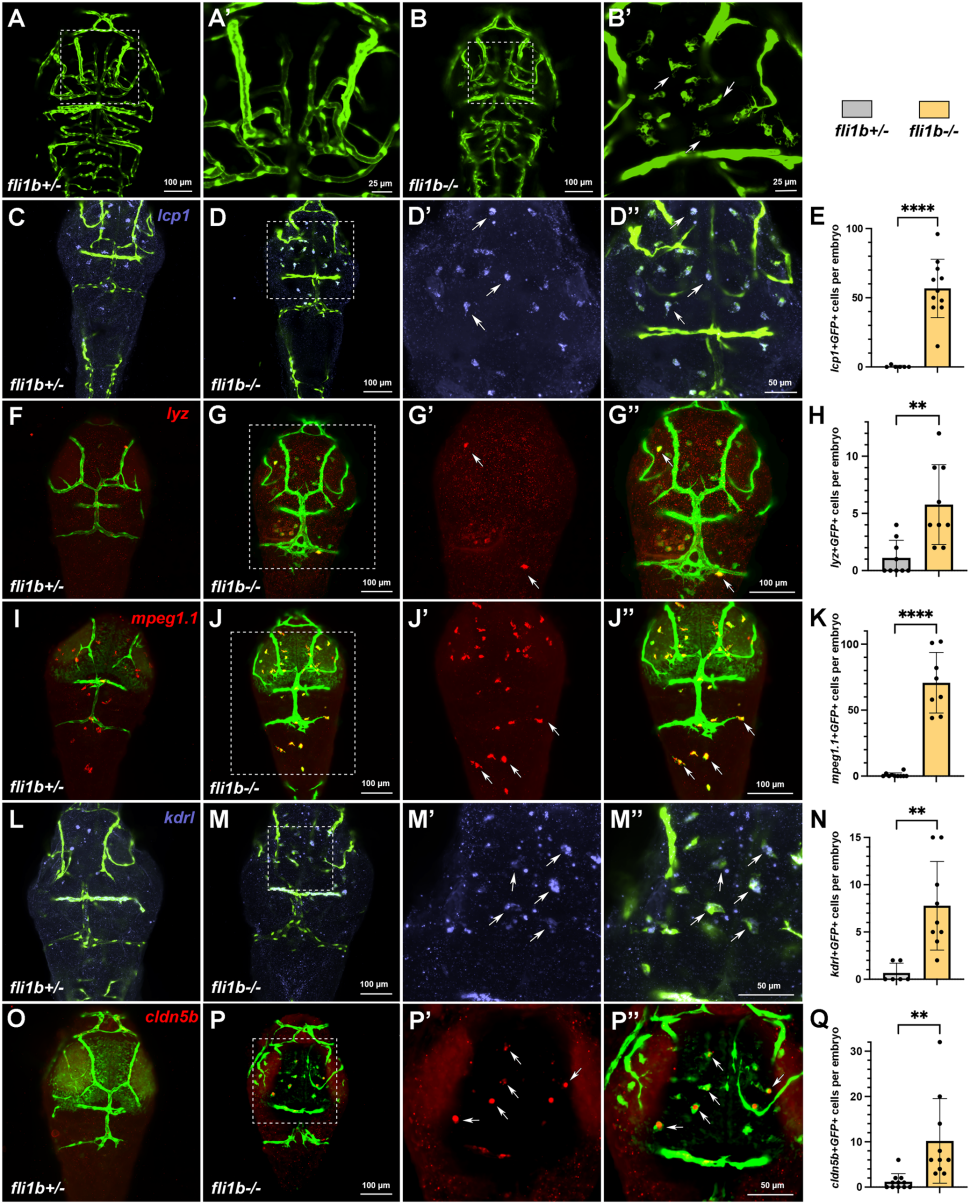
**Myeloid cells express vascular endothelial markers in *fli1b^−/−^* embryos.** (A-B′) Confocal imaging of the head region (dorsal view) of live *fli1b^+/−^* and *fli1b^−/−^* zebrafish embryos at 3 dpf. A′,B′ show higher-magnification images of A,B. Note that multiple microglia-like cells (arrows, B′) are apparent in *fli1b^−/−^* but not in *fli1b^+/−^* embryos. (C-K) HCR analysis of expression of the myeloid marker *lcp1* (blue, C-D″), the neutrophil marker *lyz* (red, F-G″) and the macrophage marker *mpeg1.1* (red, I-J″) in *fli1b^+/−^* and *fli1b^−/−^* embryos. D′,D″, G′,G″ and J′,J″ show higher-magnification views of individual and merged channels of *fli1b^−/−^* embryos in D, G and J, respectively. Arrows in Dʹ,D″,Gʹ,G″,Jʹ,J″ indicate microglia-like cells that co-express *lcp1*, *lyz* or *mpeg1.1*. (E,H,K) Quantification of GFP-positive cells that co-express *lcp1*, *lyz* or *mpeg1.1*, respectively, in the selected area of the head region of *fli1b^+/−^* and *fli1b^−/−^* embryos. (L-Q) HCR analysis of expression of the vascular endothelial marker *kdrl* and *cldn5b* in *fli1b^+/−^* and *fli1b^−/−^* embryos at 3 dpf. M′,M″,P′,P″ show higher-magnification views of M,P in *fli1b* mutant embryos. Arrows in Mʹ,M″,Pʹ,P″ indicate microglia-like cells that co-express *kdrl* and *cldn5b*. (N,Q) Quantification of GFP-positive cells that co-express *kdrl* or c*ldn5b* in the selected areas of the head region of *fli1b^+/−^* and *fli1b^−/−^* embryos. Bars show mean±s.d. **P<0.01; ****P<0.0001; Student's two-tailed unpaired *t*-test.

To determine whether these two different myeloid cell populations were present at a later stage, we analyzed myeloid marker expression and GFP fluorescence in the cranial region of *fli1b^+/−^* and *fli1b^−/−^* embryos at 3 dpf. At this stage, a striking difference in GFP expression was apparent between *fli1b^+/−^* and *fli1b^−/−^* embryos. GFP expression was restricted to vascular endothelial cells in *fli1b^+/−^* embryos, whereas there were multiple GFP^+^ macrophage-like cells with filopodia scattered in the tissues outside of the cranial vasculature in *fli1b^−/−^* embryos (Fig. 6A-B′). Time-lapse imaging demonstrated that these GFP^+^ cells actively migrated in the brain tissue in a manner similar to microglia (Movie 1). To characterize the identity of these cells, HCR was performed at 3 dpf for expression of the pan-myeloid marker *lcp1*, the neutrophil marker *lyz* and the macrophage marker *mpeg1.1*. Out of all GFP^+^ microglia-like cells quantified in a selected cranial region of *fli1b* mutant embryos, 99.2% were positive for *lcp1* (*n*=12 embryos), 100% were positive for *mpeg1.1* (*n*=10 embryos) and 19% were positive for *lyz* (*n*=9 embryos) (Fig. 6C-K). This argues that most of these migratory GFP^+^ cells are macrophages, whereas a subset of them are neutrophils, and some may co-express both markers. Because these cells were positive for GFP expression, which is linked to the *fli1b* mutation, this suggested that these cells also co-express vascular endothelial markers. Therefore, we performed HCR analysis for expression of the endothelial marker *kdrl* and *cldn5b*. Strikingly, 53% of all microglia-like GFP^+^ cells in the cranial region of *fli1b* mutants were also positive for *kdrl* expression (*n*=9 embryos), whereas 23.5% were positive for *cldn5b* expression (*n*=10 embryos). Almost none or very few such cells were observed in *fli1b^+/−^* embryos, in which *kdrl* and *cldn5b* expression was restricted to vascular endothelial cells (Fig. 6L-Q). These results argue that many myeloid cells co-express endothelial markers in *fli1b^−/−^* embryos.

### Single-cell transcriptomic analysis of *fli1b^−/−^* embryos

**Fig. 7.**
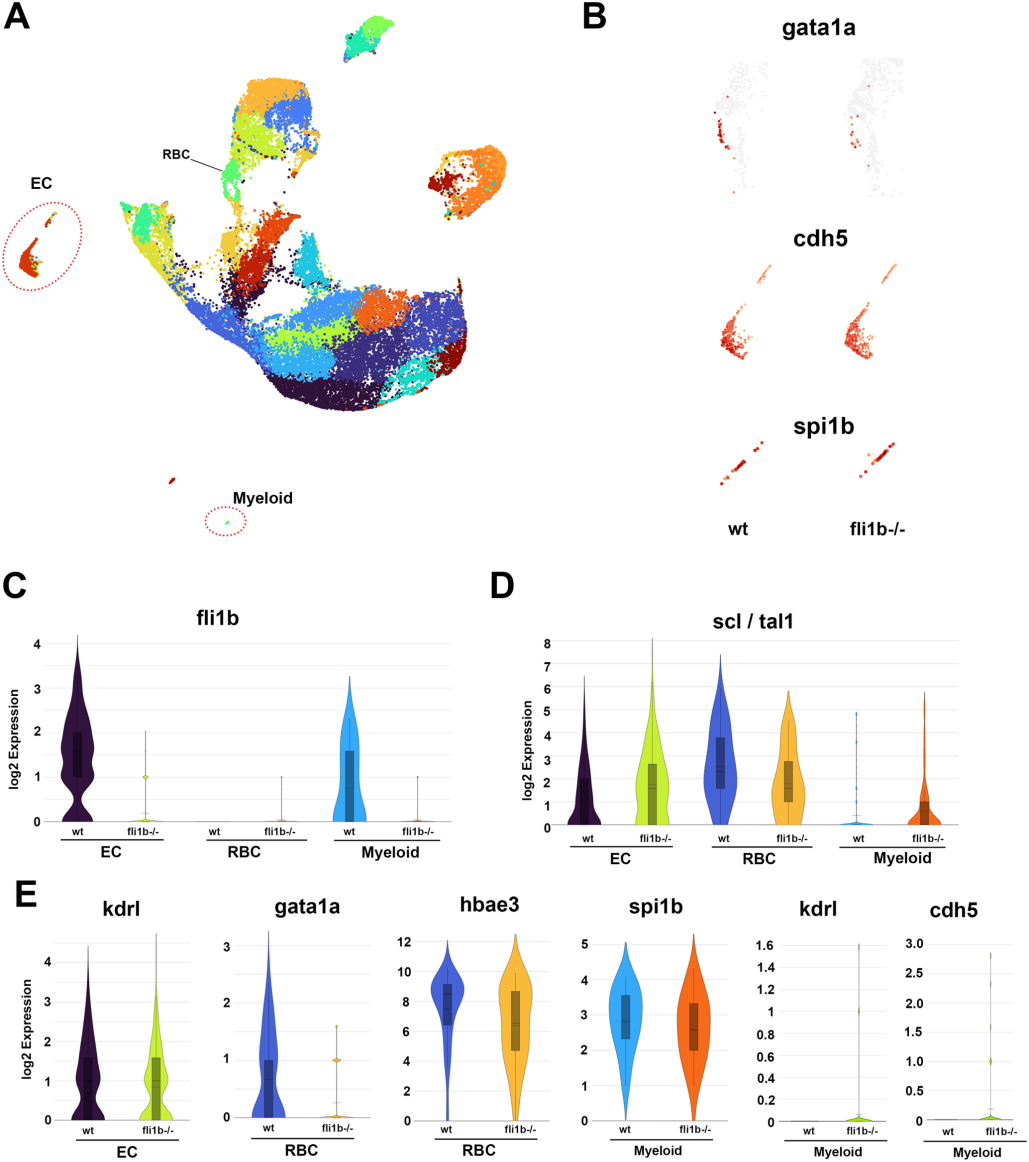
**Single-cell RNA-seq analysis of *fli1b* mutant and wt embryos at 23-24 hpf.** (A) Uniform manifold approximation and projection (UMAP) plot showing aggregated cell clusters from wt and *fli1b* mutant embryos, obtained by the corresponding in-crosses of sibling parents. Vascular endothelial cell (EC), red blood cell (RBC) and myeloid cell populations were identified based on marker expression (red dotted circles). (B) Feature plots for the RBC marker *gata1a*, the EC marker *cdh5* and the myeloid marker *spi1b* in RBCs, endothelial and myeloid clusters, respectively, of wt and *fli1b* mutant embryos. (C,D) Violin plots of *fli1b* (C) and *scl* (D) expression in EC, RBC and myeloid populations. (E) Violin plots of *kdrl* expression in ECs, *gata1a* and *hbae3* expression in RBCs, and *spi1b*, *kdrl* and *cdh5* expression in myeloid populations. Note that *gata1a* and *hbae3* expression was reduced in *fli1b^−/−^* embryos, whereas *kdrl* and *spi1b* expression showed no significant change. *kdrl* and *cdh5* expression is apparent in a subset of myeloid cells in *fli1b* mutants but not in wt embryos.

To characterize transcriptomic changes in different cell populations, we performed single-cell RNA-sequencing (RNA-seq) analysis. Homozygous *fli1b^−/−^* mutant and sibling wt parents were in-crossed to generate populations of *fli1b^−/−^* and wt embryos. All cells were disaggregated at the 23 to 24 hpf stage and subjected to single-cell RNA-seq profiling using the Chromium (10× Genomics) platform. After the initial filtering steps, 12,957 cells were identified in wt embryos and 15,271 cells were identified in *fli1b* mutant embryos. Unsupervised clustering of aggregated wt and *fli1b* cells identified 26 distinct clusters (Fig. 7A). We focused further analysis on vascular endothelial cell, RBC and myeloid cell clusters, which were identified based on expression of the marker genes *cdh5*, *gata1a* and *spi1b*, respectively (Fig. 7A,B). To identify changes in gene expression, we performed differential expression analysis between endothelial cell, RBC and myeloid cell populations in *fli1b* mutant and wt embryos. Differential expression analysis in RBCs identified approximately 20 significantly downregulated genes in *fli1b* mutants, which included the myeloid markers *spi1b*, *lcp1* and *coro1a*, and RBC-specific markers, such as *urod* and *alas2* (Table 1). Many other RBC markers, including the globin genes *hbae4*, *hbae3*, *hbbe3* and *hbbe2*, as well as *gata1a* also showed greater than 2-fold reduction in *fli1b* mutants, although they did not reach statistical significance (*P*<0.05) (Table 1, Fig. 7E). It is known that *spi1b* and other myeloid markers are also expressed in early erythrocytes (Bennett et al., 2001; Herbomel et al., 1999; Lieschke et al., 2001), which explains why their downregulation was observed in the RBC cluster. Differential expression analysis in vascular endothelial cells identified 19 significantly downregulated genes, including *plvapb* and *glula* (Table 2)*.* The top endothelial downregulated gene was *fli1b* itself, expression of which is known to be inhibited in *fli1b* mutants (Table 2, Fig. 7C) (Craig et al., 2015). Violin plots show that *fli1b* expression was high in both endothelial and myeloid cells in wt embryos, and very low in *fli1b* mutant embryos (Fig. 7C). However, most endothelial-specific genes, including *kdrl*, were not significantly affected in *fli1b* mutant embryos (Fig. 7E). Differential expression analysis between *fli1b* mutant and wt embryos in myeloid cells did not identify any significantly downregulated genes, and myeloid *spi1b* expression was not significantly affected (Fig. 7E). By analyzing the expression of selected marker genes, we noticed that some vascular endothelial genes, including *kdrl* and *cdh5*, showed expression in a subset of myeloid cell population in *fli1b^−/−^* mutants, whereas they were absent from myeloid cells in wt embryos (Fig. 7E). This is consistent with our observations of endothelial marker expression in myeloid cells using HCR analysis. Single-cell RNA-seq is known to have limited sensitivity and to be prone to ‘dropouts’ of low-expressing genes (Stegle et al., 2015), which could be the reason why only few myeloid cells show endothelial cell marker expression in this dataset. Intriguingly, *scl* expression was increased in endothelial cell and myeloid cell populations in *fli1b* mutants and reduced in RBCs (Fig. 7D). Reduction of *scl* expression in RBCs correlates with reduced erythroid differentiation. Its increase in endothelial cells could indicate that endothelial cells have a mixed endothelial-hematopoietic identity or that there is an increased population of angioblasts, which have higher *scl* expression, compared to differentiated endothelial cells. In summary, single-cell transcriptomic analysis correlates with the results of HCR analysis and indicates reduced erythroid differentiation and mixed cell identities in myeloid cells of *fli1b* mutant embryos.

**Table 1.** Differential gene expression analysis in red blood cells between *fli1b^−/−^* and wild-type embryos at 23 hpf

Gene symbol	Log_2_(fold change)	*P*-value	Expression
*zgc:173709*	−4.6022105	6.08×10^−10^	Unknown
*lcp1*	−6.2596089	4.03×10^−9^	Myeloid
*ENSDARG00000093877*	−6.5864862	1.11×10^−8^	Unknown
*mfap4.1*	−8.2780815	9.25×10^−8^	Macrophages
*mfap4.2*	−8.5377469	9.16×10^−7^	Macrophages
*spi1b*	−3.9780129	9.59×10^−7^	Myeloid
*srgn*	−6.6461069	1.93×10^−5^	Neutrophils
*ccl34a.4*	−8.9543168	4.46×10^−5^	Macrophages
*arhgdig*	−3.0542279	5.70×10^−5^	Unknown
*coro1a*	−2.8587139	0.00013966	Myeloid
*ENSDARG00000112540*	−7.4179192	0.00014733	Unknown
*ENSDARG00000075664*	−7.5963339	0.0001811	Unknown
*rac2*	−2.5798427	0.00048154	Neutrophils
*f13a1b*	−2.0735466	0.00211112	Macrophages
*lyz*	−7.7218321	0.00223547	Neutrophils
*gpx1a*	−2.0572884	0.00359811	Multiple tissues
*pfn1*	−1.8145141	0.00460611	Blood
*urod*	−1.4434099	0.01169871	RBCs
*prdx5*	−1.4899909	0.03011707	Macrophages
*alas2*	−1.3443406	0.05399074	RBCs
*hmbsb*	−1.4194329	0.05487578	RBCs
*blf*	−1.4699783	0.0634061	RBCs
*hbae3*	−1.0871172	0.33848699	RBCs
*hbbe3*	−1.0226347	0.39089267	RBCs
*hbbe2*	−1.0305587	0.3919518	RBCs

Top downregulated genes in *fli1b* mutants are shown. Expression patterns are based on https://zfin.org/. RBCs, red blood cells.

**Table 2.** Gene expression changes in endothelial cells between *fli1b* mutant and wild-type embryos

Gene symbol	Log_2_(fold change)	*P*-value
*fli1rs/fli1b*	−3.3314987	2.47×10^−29^
*plvapb*	−1.489652	1.13×10^−5^
*glula*	−1.639338	1.19×10^−5^
*ENSDARG00000069998*	−1.3855526	0.00047175
*itga1*	−1.1728488	0.00355038
*ctsla*	−1.0527212	0.00355038
*ENSDARG00000021987*	−1.0940732	0.00511676
*txnipa*	−1.0004508	0.00948293
*dab2*	−0.944394	0.01623027
*mafbb*	−0.9511739	0.01717148
*nrp2b*	−1.0032883	0.01873688
*gpr182*	−0.9354846	0.01880407
*calcrla*	−0.9845305	0.0250077
*stab2*	−0.8975473	0.03193187
*prcp*	−0.8878236	0.03893257
*ap1s3b*	−0.9081781	0.03911538
*scarf1*	−0.8637475	0.04041173
*hyal2*	−0.9067251	0.04579256
*sypl2a*	−0.8507553	0.0491779

All genes with *P*<0.05 are shown.

## DISCUSSION

In this study, we demonstrate the requirement for zebrafish *fli1b* in regulating embryonic hematopoiesis, including the emergence of the primitive erythroid and myeloid lineages as well as the specification of HSPCs. Zebrafish *fli1b* mutant embryos showed reduced number of RBCs and diminished expression of erythroid-specific markers, including *gata1* and *hbae3.* The transcription factor Scl is the master regulator of hematopoietic differentiation and functions upstream of *gata1* during erythropoiesis. Previous studies have shown that zebrafish *scl* knockdown results in the inhibition of hematopoiesis and reduction of vasculogenesis (Dooley et al., 2005; Juarez et al., 2005; Patterson et al., 2005). Our results show that *scl* expression in erythroid progenitors was reduced in *fli1b* mutant embryos. Furthermore, induction of *scl* expression partially restored erythroid development in *fli1b* mutant embryos. These results argue that *fli1b* regulates primitive erythropoiesis through *scl* expression. These findings agree with previous studies in mouse embryoid bodies and fetal liver, which have demonstrated that *Fli1* and *Scl* can positively regulate expression of each other (Pimanda et al., 2007).

**Fig. 8.**
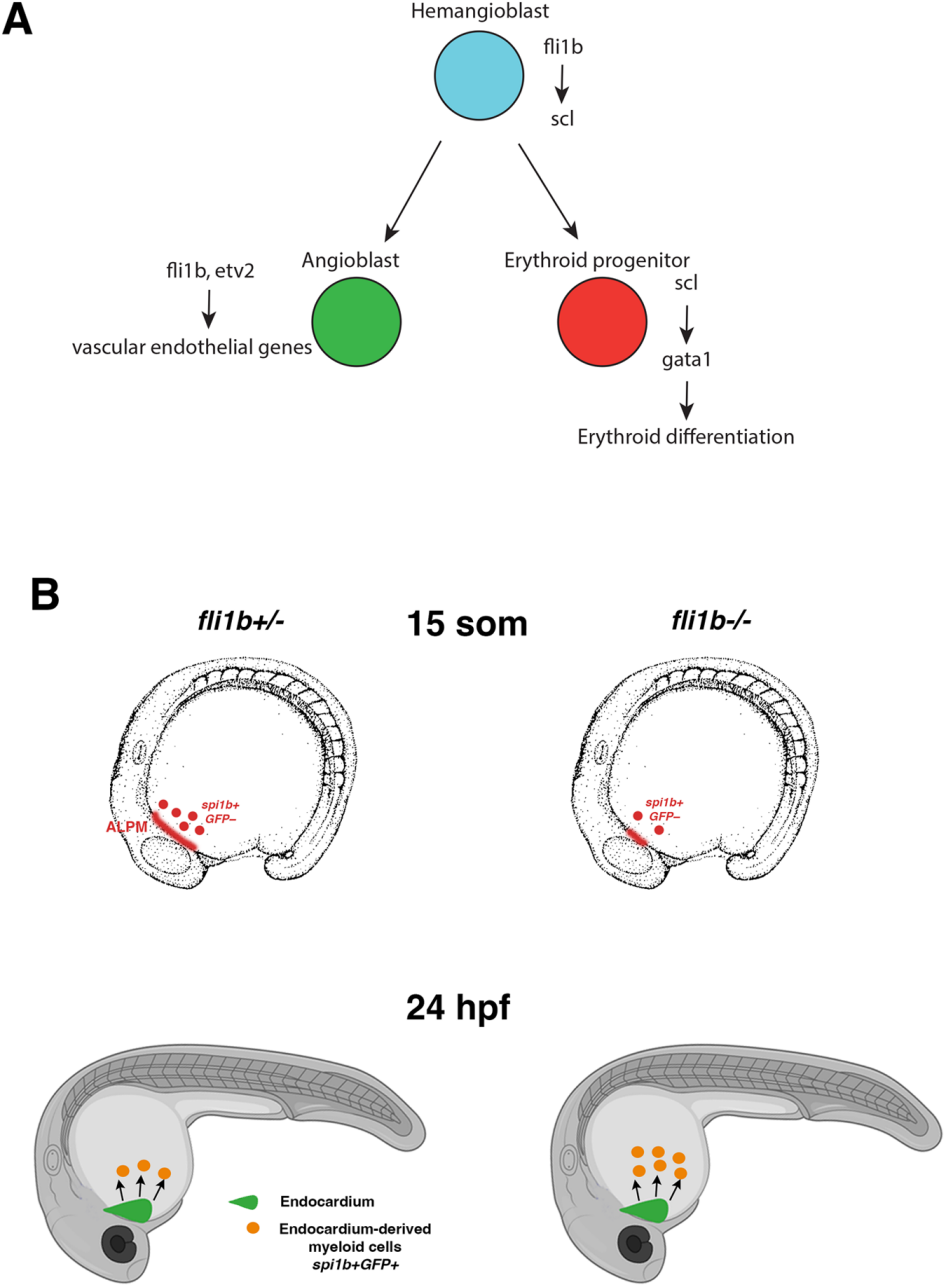
**A proposed model for the role of *fli1b* during the emergence of hematopoietic lineages.** (A) *fli1b* is expressed in the common precursor of vascular endothelial cells and erythrocytes, the hemangioblast, where it upregulates *scl* expression. After the angioblast and erythroid lineages separate, *fli1b* is maintained only in angioblast/vascular endothelial progenitor cells, where it promotes endothelial differentiation together with *etv2*, whereas *scl* promotes the erythroid differentiation program. (B) The role of *fli1b* in myeloid differentiation. *fli1b* is required for *spi1b* expression in the earliest myeloid progenitors, which emerge from the anterior lateral plate mesoderm (ALPM) prior to the 15-somite stage. At approximately 24 hpf, the second wave of myeloid cells emerges from the endocardium. Endocardial-derived myelopoiesis is increased in *fli1b* mutants.

Although *fli1b* mutants show strong downregulation of primitive erythropoiesis, *fli1b* appears to have little to no expression in erythroid cells, and its expression is restricted to vascular endothelial cells. It is possible that *fli1b* function is required non-cell-autonomously in vascular endothelial cells. However, we consider this possibility unlikely because *fli1b* is a transcription factor, which typically function cell-autonomously. In addition, RBCs appear to differentiate largely normally even in the absence of vascular endothelial cells, as observed in *etv2* mutant or knockdown embryos (Sumanas and Lin, 2006). A more likely explanation is that *fli1b* regulates hematopoiesis at the hemangioblast stage, where it induces *scl* expression. However, *fli1b* expression is not maintained in RBCs and is restricted to endothelial cells at later stages (Fig. 8A).

Although *fli1b* mutants showed strong inhibition of erythroid differentiation, the phenotype was quite variable between different embryos. This could be due to redundancy with another ETS factor, such as *fli1a*, that is expressed in both vascular endothelial and hematopoietic cells (Brown et al., 2000). To date, no hematopoietic defects have been described in zebrafish *fli1a* knockdown embryos and no in-depth studies of *fli1a* mutant phenotype have been performed.

In addition to defective primitive hematopoiesis, *fli1b* mutants also display loss or reduction of HSPC formation, as evidenced by the reduced *runx1* and *cmyb* expression. HSPCs emerge from the hemogenic endothelium within the dorsal aorta (Bertrand et al., 2010; Kissa and Herbomel, 2010). However, vasculogenesis and dorsal aorta formation do not appear to be affected in *fli1b* mutants, based on our previous studies (Craig et al., 2015). This suggests that *fli1b* mutants have a specific defect in hemogenic endothelium formation, possibly through misregulation of *scl* expression, which plays a critical role in HSPC specification (Zhen et al., 2013).

Our recent study has demonstrated that primitive myeloid cells emerge from two distinct sites in zebrafish embryos (Gurung et al., 2024). Although the earliest myeloid cells, which include macrophages and neutrophils, originate from the ALPM at the 10- to 15-somite stages, an independent population of myeloid cells emerges from the endocardium at the 22 to 28 hpf stages (Fig. 8B) (Gurung et al., 2024). These cells contribute mostly to the neutrophil lineage, although a small percentage can also contribute to the macrophage lineage. They can be distinguished by early expression of vascular endothelial/endocardial markers such as *kdrl*, which are absent from ALPM-derived myeloid cells (Gurung et al., 2024). However, this endothelial marker expression in wt embryos is transient and is no longer observed at 48 hpf or later stages in the endocardial-derived myeloid cells. Intriguingly, *fli1b* mutants exhibit opposing effects on the two populations of myeloid cells. GFP-negative cells, which likely correspond to the ALPM-derived myeloid cells, are reduced in 24 hpf embryos. This reduction of ALPM-derived myeloid cells was also confirmed by the analysis of *spi1b* expression at the 15-somite stage, which was strongly reduced in *fli1b* mutants. In contrast, the GFP^+^ population, which likely corresponds to the endocardial-derived myeloid cells, was significantly expanded in *fli1b* mutant embryos. This correlates with the higher number of *spi1b*-positive cells observed in the endocardium of *fli1b* mutants. The mechanism of increased myeloid cell production in the endocardium is not clear. It may occur as a compensatory response due to the reduction in ALPM-derived myeloid cells. A similar compensation by endocardial-derived myeloid cells was also observed in *etv2* mutant embryos, which also exhibit loss of ALPM-derived myeloid cells (Gurung et al., 2024).

Surprisingly, GFP expression in myeloid cells was apparent in *fli1b* mutants at 3 dpf, which was largely absent in *fli1b^+/−^* embryos. Because GFP expression is linked to *fli1b* mutation, this suggests that *fli1b* promoter activity, which is normally restricted to vascular endothelial cells at 3 dpf, was also pronounced in myeloid cells in *fli1b* mutants. Indeed, our results show that other vascular endothelial markers, such as *kdrl* and *cldn5b*, were expressed in the myeloid cells in *fli1b* mutants, whereas they were absent from the myeloid cells in *fli1b^+/−^* embryos. Although endocardial-derived myeloid cells show residual expression of endothelial markers at 24 hpf, such expression is no longer apparent at 2-3 dpf (Gurung et al., 2024). Therefore, the endocardial origin cannot explain prolonged expression of GFP (linked to *fli1b* mutation) and vascular endothelial markers in *fli1b* mutants. It is possible that these cells are arrested in a hemangioblast-like state and, even though they commit to myeloid differentiation, they are unable to downregulate vascular endothelial markers. This suggests that *fli1b* is important for cells to transition from hemangioblast to hematopoietic lineages.

Global transcriptomic single-cell analysis confirmed the results obtained using HCR. It showed reduced expression of *scl*, *gata1* and *hbae3* and many other RBC markers in erythroid cells of *fli1b* mutants. Although overall myeloid *spi1b* expression was unaffected, there was a small but notable expression of vascular endothelial markers apparent in myeloid cells, consistent with HCR results. However, only a relatively small number of genes were significantly affected in *fli1b* mutants. This is likely due to the methodology used in this study, which involved a whole-embryo disaggregation strategy that resulted in identification of rather small populations of hematopoietic cells and, therefore, limited the statistical significance of observed changes.

Similar to zebrafish *fli1b* mutants, mouse Fli1 has been implicated in regulating myeloid differentiation and *Spi1* expression (Masuya et al., 2005; Starck et al., 1999; Suzuki et al., 2013). Differently from the zebrafish *fli1b* mutants, primitive erythropoiesis does not appear to be greatly reduced in mouse *Fli1* mutants (Spyropoulos et al., 2000). In contrast, *Fli1* overexpression reduces erythroid differentiation in mammalian cells, suggesting that *Fli1* negatively regulates erythropoiesis in mouse embryos (Pereira et al., 1999; Tamir et al., 1999). It is possible that mouse *Fli1* mutant embryos exhibit slight defects in erythroid differentiation, which have not been observed or well characterized yet. However, it is also possible that there are species-specific differences between transcription factors. In mice, the related ETS transcription factor Etv2 plays a critical role in both hematopoiesis and vasculogenesis, and mouse Etv2 mutants show loss of hematopoiesis, including deficient erythroid differentiation (Ferdous et al., 2009; Lee et al., 2008). However, zebrafish *etv2* knockdown embryos show only loss of endothelial and myeloid lineages, whereas erythropoiesis is largely unaffected (Sumanas et al., 2008; Sumanas and Lin, 2006). It is possible that zebrafish Fli1b has a requirement in erythropoiesis, which is equivalent to the role of Etv2 in murine embryos.

In summary, this study has identified new roles for the transcription factor Fli1b in regulating hematopoiesis. These results will promote our understanding of molecular mechanisms that regulate hematovascular differentiation during development and may also contribute to the development of improved strategies to generate hematopoietic stem and progenitor cells for regenerative medicine.

## MATERIALS AND METHODS

### Fish lines and embryos

All studies were performed according to the animal protocols approved by the University of South Florida Institutional Animal Care and Use Committee. Male and female adults between 3 months and 2 years of age were used for mating to acquire embryos for experiments. Experiments utilizing embryos during the stages of somitogenesis were performed with incubation at 25°C. All other embryos used for analysis were incubated at 28.5°C in embryo medium; embryos analyzed past 24 hpf were also treated with 0.003% 1-phenyl-2-thiourea (PTU; Millipore Sigma) to prevent pigment formation. The previously established *fli1b^tpl50Gt^* line (further labeled as *fli1b^+/−^* or *fli1b^−/−^*) was used in the study (Craig et al., 2015). The targeting vector contains Gal4 and UAS:GFP elements, and results in vascular endothelial GFP expression when inserted in *fli1b* locus (Craig et al., 2015). *fli1b^−/−^* mutant embryos were obtained by the cross of homozygous *fli1b^−/−^* parents, whereas control *fli1b^+/−^* embryos were obtained by mating *fli1b^+/−^* adults (which were siblings of *fli1b^−/−^* adults) to wt AB line and selecting embryos with GFP fluorescence. In some experiments, wt *Tg(fli1a:GFP)^y1^* (Lawson and Weinstein, 2002) controls were used, as noted in the text or the figure legend. Other lines used included: *Tg(kdrl:GFP)^s843^* (Jin et al., 2005), *Tg(kdrl:mCherry)^ci5^* (Proulx et al., 2010) and *TgPAC(scl:dsRed)* (Zhen et al., 2013).

### WISH

WISH was performed as previously described (Jowett, 1999). RNA probes were synthesized using T3, T7 or SP6 RNA Polymerases (Promega). RNA probes used were for *gata1* (Detrich et al., 1995), *hbbe3* (Brownlie et al., 2003), *scl* (Liao et al., 1998), *runx1* (Galloway et al., 2005) and *cmyb* (Thompson et al., 1998). Prior to WISH, embryos were fixed in BT-Fix (4% paraformaldehyde in 1× PBS) at 4°C overnight, then dehydrated through sequential ethanol series and stored at −20°C in 100% ethanol. For WISH, embryos were sequentially rehydrated and washed three times with PBT (1× PBS, 0.2% BSA, 0.2% Tween 20). Embryos fixed at 24 hpf and 3 dpf were digested with proteinase K (10 µg/ml; Thermo Fisher Scientific) for 5 min and 30 min, respectively, at room temperature. Following this, embryos were washed with PBT and refixed with BT-Fix for 20 min, then washed three times for 5 min with PBT at room temperature. Embryos were then incubated in prewarmed prehybridization buffer [prehyb: 50% formamide, 5× sodium citrate buffer ( SSC) 50 µg/ml heparin, 5 mM EDTA, 0.5 mg/ml torula yeast RNA (Millipore Sigma), 9.2 mM citric acid, 0.1% Tween 20] for 2 h at 65°C while shaking, incubated overnight with a probe solution in prehyb at 65°C, and subsequently washed with the following solutions at 65°C: 75% prehyb and 25% 2× SSC; 50% prehyb and 50% 2× SSC; 25% prehyb and 75% 2× SSC; 2× SSC; and twice with 0.2× SSC. Subsequent room temperature washes included: 75% 0.2× SSC and 25% PBT; 50% 0.2× SSC and 50% PBT; 25% 0.2× SSC and 75% PBT; and 100% PBT. After washes, embryos were incubated overnight in 1× PBT with 2% lamb serum and a 1:4000 dilution of anti-digoxigenin-AP antibody (Sigma-Aldrich, 11093274910). Embryos were subjected to six washes in PBT and were then incubated in AP buffer (100 mM NaCl, 50 mM MgCl_2_, 100 mM Tris-Cl, pH 9.5, 0.1% Tween 20) containing 4-nitrotetrazolium blue chloride (NBT, 0.225 mg/ml; Millipore Sigma) and 5-bromo-4-chloro-3-indolyl phosphate disodium salt (BCIP, 0.175 mg/ml; Millipore Sigma). Staining was ended with additional washes in PBT, then embryos were incubated in BT-Fix for 2 h. Embryos were scored into groups and subsequently mounted in slide chambers using 0.6% low-melting agarose. Stained embryos following WISH were imaged using a 10× objective on an AxioImager Z1 compound microscope from Zeiss, equipped with an Axiocam ICC3 color camera, or a Nikon Eclipse Ni-E microscope.

### O-dianisidine heme staining

Live embryos at 2 and 3 dpf were developed with o-dianisidine staining solution [6 mg o-dianisidine-chloride, 6 ml water, 33 µl 3 M sodium acetate (pH 5), 4 ml ethanol, 186 µl 35% hydrogen peroxide] in the dark for 1 h. Then, embryos were washed with PBST (1× PBS, 0.2% Tween 20), fixed with BT-Fix for 2 h at room temperature, and washed with PBST three times for 5 min.

### qPCR analysis

Batches of 20-25 embryos were frozen on dry ice at the 20-somite stage. They were then homogenized in lysis buffer from the RNAqueous 4-PCR Kit (Thermo Fisher Scientific) with a 23-gauge needle, and extraction of RNA from embryos was performed using the same RNAqueous 4-PCR kit (Thermo Fisher Scientific). cDNA synthesis was performed using the SuperScript VILO cDNA Synthesis Kit or the Superscript III cDNA synthesis kit (Thermo Fisher Scientific). Quantitative real-time PCR was performed using PowerUp SYBR Green Master Mix (Applied Biosystems) in a Cielo Real-Time PCR System (Azure Biosystems). Quantification was performed using the relative standard curve method; controls were assigned a value of 1 and experimental values were computed by the software based on C_T_ values, relative to the control standard curve. For each experiment, two to three replicates were performed with duplicates in each run, and *ef1α* was used as an endogenous control. The relative quantity of cDNA for each sample was normalized to the value of *ef1α*. Data were analyzed using the Azure Cielo Manager Software (Azure Biosystems). Primer sequences are listed in Table S1.

### Fluorescence *in situ* hybridization (FISH)

FISH was performed using HCR (Choi et al., 2018). HCR v3.0 probes for *gata1*, *fli1b*, *scl*, *lcp1*, *kdrl*, *lyz*, *mpeg1.1* and *spi1b* were obtained from Molecular Instruments, Los Angeles, CA, USA. HCR was performed using embryos fixed overnight with BT-Fix at 4°C, dehydrated in sequential ethanol series and stored at −20°C. Embryos were rehydrated and washed three times with PBT. A prehybridization step was done using hybridization buffer (30% formamide, 5× SSC, 9 mM citric acid at pH 6.0, 0.1% Tween 20, 50 μg/ml heparin, 1× Denhardt's solution and 10% dextran sulfate) for 30 min at 37°C. Following this, each probe (2 pmol) was combined with 500 µl of hybridization buffer and incubated overnight gyrating at 37°C. Following incubation, embryos were washed four times for 15 min with 30% formamide, 5× SSC, 9 mM citric acid at pH 6.0, 0.1% Tween 20 and 50 µg/ml heparin at 37°C. This was then followed with three 5 min washes with 5× SSCT (5× SSC and 0.1% Tween 20) at room temperature and a 30 min incubation in amplification buffer (5× SSC, 0.1% Tween 20 and 10% dextran sulfate) at room temperature. Hairpin probes (30 pmol each), fluorescently labeled through snap cooling of 3 µM stock solution, were added to the embryos in amplification buffer and incubated overnight at room temperature in the dark. Next, samples were washed five times with 5× SSCT. Embryos were mounted in 0.6% low-melting agarose and imaged using a Nikon A1R HD confocal microscope using a 20× objective.

### MO injection

A previously validated translation-blocking *scl* MO (5′-AATGCTCTTACCATCGTTGATTTCA-3′) obtained from Gene Tools (Philomath, OR, USA) was used (Dooley et al., 2005; Gurung et al., 2024). Embryos were injected into the yolk at the one-cell stage with 7.5 ng (per embryo) of this *scl* MO.

### RNA overexpression

*scl* mRNA (Addgene, Watertown, MA, USA) was prepared by NotI linearization of the *scl-pCS2+* expression vector (Mattonet et al., 2022), followed by transcription using SP6 mMessage Machine kit (Thermo Fisher Scientific). At the one-cell stage, 100 pg of *scl* mRNA was injected into zebrafish embryos. Embryos were then fixed at 22 hpf for FISH.

### Single-cell RNA-seq analysis

*fli1b^−/−^* and wt embryos were obtained by the corresponding in-crosses of sibling parents that were either homozygous mutant (*fli1b^−/−^*) or wt (*fli1b^+/+^*)*.* Approximately 40 embryos from each sample were manually dechorionated at approximately 23-24 hpf, then transferred into 1.5 ml tubes. Whole embryos were then dissociated into a single-cell suspension by cold protease tissue dissociation protocol (Potter and Potter, 2019). All steps were performed on ice. Embryo water was removed and deyolking buffer was added (55 mM NaCl, 1.8 mM KCl and 1.25 mM NaHCO_3_ in 1× PBS). Embryos were pipetted up and down until the yolk was dissolved, then centrifuged at 300 ***g*** for 1 min. The supernatant was aspirated and embryos were resuspended in 0.5× Danieau buffer. This step was repeated and, after the supernatant was aspirated, *Bacillus lichenformis* enzyme mix [1× fetal bovine serum (FBS; Millipore Sigma) in PBS, 0.5 mM of EDTA, 125 U/ml DNase 1 (Thermo Fisher Scientific), 10 mg/ml *Bacillus lichenformis* (Millipore Sigma)] was prepared and added to embryos. Embryos were incubated on ice for 20 min and titrated 15 times every 2 min. Cell dissociation was monitored under microscope and, once completed, centrifugation was performed at 1200 ***g*** for 5 min. The supernatant was discarded and the pellet was resuspended in 10% FBS in PBS. Cells were then passed through 20-µm strainer, then washed with 500 µl of 10% FBS. Cells were then pelleted again at 1200 ***g*** for 5 min, washed once with 1 ml of 10% FBS, and centrifuged at 1200 ***g*** for 5 min. Cells were resuspended in 250 µl of 10% FBS, then counted using a hemocytometer. A suspension of approximately 16,000 cells (for each genotype) was loaded onto the 10× Genomics Single Cell 3′ chip at the University of South Florida Genomics Core. Samples were sequenced on an Illumina NextSeq 2000 instrument (Illumina, San Diego, CA, USA). The raw .fastq files obtained from the sequencing core were then mapped to the *Danio rerio* genome (Zv11) to generate single-cell feature counts using Cell Ranger version 8.0 (10× Genomics). Datasets from wt and *fli1b* mutant embryos were aggregated using the Cell Ranger ‘aggreg’ function. Two rounds of sequencing were performed for each sample, and the sequence files were aggregated in Cell Ranger for the final analysis. Cell clustering and data analysis were performed using Loupe Browser version 8.0 (10× Genomics). Cells were filtered based on unique molecular identifiers (2213-30159 range), expressed genes (799-4873) and mitochondrial unique molecular identifiers (<5%). This resulted in the identification of 28,228 cells (12,957 wt and 15,271 *fli1b^−/−^* cells), which clustered into 26 individual clusters. The myeloid cell cluster was not identified in the initial clustering and was then subset manually based on marker gene expression using Loupe Browser.

### Imaging and cell quantification

Live or fixed embryos were mounted in 0.6% low-melting point agarose. 0.002% tricaine (Sigma-Aldrich, SKU#A5040) was added for live-embryo imaging. Imaging was performed on a Nikon Eclipse confocal microscope. Images were processed using the ‘denoiseAI’ function (NIS Elements, Nikon) to reduce the noise. Selected slices were used to create a maximum-intensity projection in NIS Elements AR software (Nikon). All image panels were created using Adobe Photoshop CC, and image levels were adjusted in Adobe Photoshop CC to optimize brightness and contrast. The same adjustments were performed on control and experimental embryos in all cases.

To quantify the fluorescence intensity of *scl* expression at the 15-somite stage (Fig. 3A-D), the average integrated density was measured using Fiji/ImageJ within small areas of ten selected representative cells in each embryo. The average background value was then subtracted for each embryo.

To quantify the intensity of *fli1b* expression in control and *scl* MO-injected embryos (Fig. 3I-K), the rectangle tool in Fiji was used to select the measured area that constituted most of the *fli1b* expression domain in the trunk region above the yolk extension. The width and length of the rectangle were kept constant between all embryos analyzed. The integrated density was measured and the background was subtracted. All measurements in *scl* MO-injected embryos were then normalized to the average values in control embryos.

Fluorescence intensity quantification in *scl* mRNA injection experiments (Fig. 3L-T) was performed using the custom shape tool in Fiji software to make a tight area encircling *gata1* expression along the yolk extension of the embryo. The integrated intensity value was measured in the selected area. The background was measured using the rectangle tool and adjusted according to the area measured for *gata1* expression. Then, it was subtracted from the integrated density to calculate the final value.

### Statistical methods

Sample size and number of replicates are indicated in the main figure or figure legends for each specific analysis. All experiments have been replicated at least twice. Calculation of mean±s.d., two-tailed unpaired Student's *t*-test, Fisher's test and plotting of graphs were performed in GraphPad Prism.

## Supplementary Material

10.1242/biolopen.061948_sup1 Supplementary information
