# Supplementary material for: The zebrafish ETS transcription factor Fli1b functions upstream of Scl/Tal1 during embryonic hematopoiesis
Source: Biol Open. 2025 Apr 9;14(4):bio061948. doi: 10.1242/bio.061948 (PMC12010910; doi:10.1242/bio.061948)
Supplement: Supplementary information [file biolopen-14-061948-s1.pdf]

Table S1. qPCR primer sequences.

gata1a_F	CCAGAGCCGAGCCTCGTCAT	gata1a_R	GCTTCCACTTCCACTCATGG
hbae3_F	CCTGCCCACAGCTAACCAAG	hbae3_R	CACCAGCAGATTGTGGTTGA
scl_F	GATGGCGCAGCTCAACGTC	scl_R	GGATTCGTCGTTGCGGGATG
fli1b F	GACCAAAGTGCACGGCAAACGC	fli1b R	GTGTTCAAGTGAGTGTGAGTGCTGG
ef1a F	TCACCCTGGGAGTGAAACAGC	e1a R	ACTTGCAGGCGATGTGAGCAG

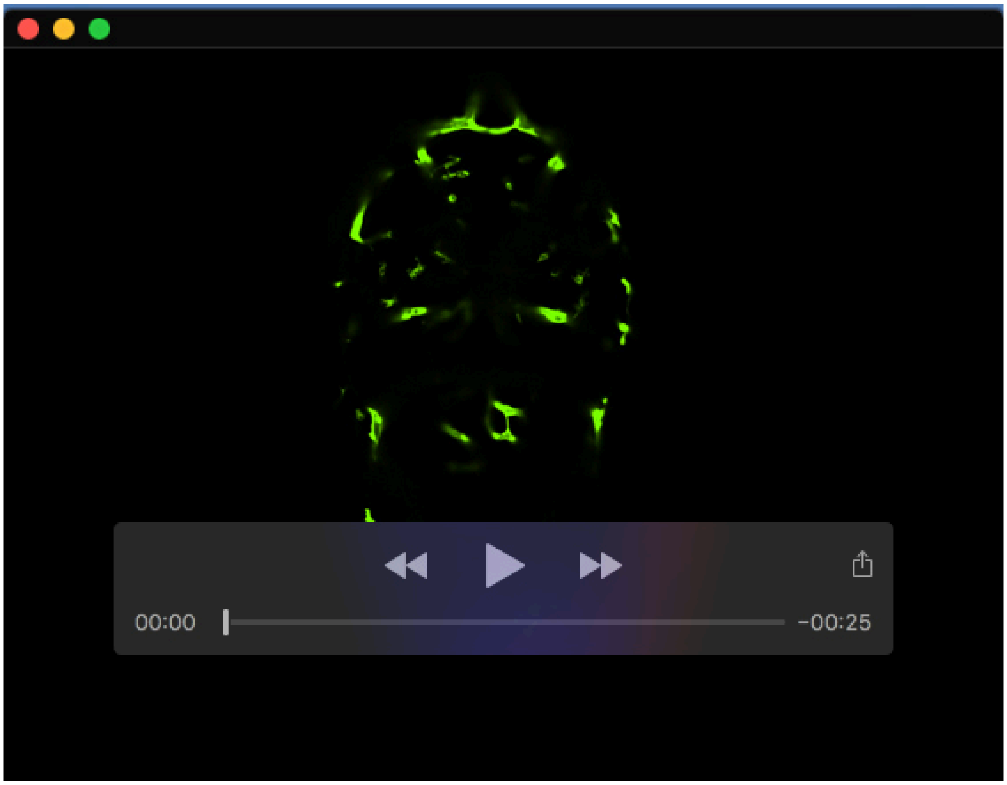

Movie 1. Time-lapse video imaging microglia-like cells in *fli1b* mutants at 3 dpf. Dorsal view of the cranial region, anterior is to the top. Maximal intensity projection is shown, each frame was acquired approximately every 4 minutes for about 2 hours total.

Biology Open • Supplementary information
